# The influence of semantic relevance on the discernment of product appearance and function

**DOI:** 10.1186/s40359-021-00632-4

**Published:** 2021-09-03

**Authors:** Ching-Yi Wang, Yu-Er Lin

**Affiliations:** 1 Present Address: Department of Creative Product Design, Asia University, No. 500, Lioufeng Rd., Wufeng, Taichung City, 41354 Taiwan; 2 Department of Industrial Design, Tatung University, No. 40, Chungshan North Rd., Section 3, Taipei City, 104 Taiwan

**Keywords:** Event-related potentials (ERPs), N600, Semantic, Figurative product, Metaphor

## Abstract

**Background:**

This study investigated the impact of semantic relevance on the ability to comprehend the appearance and function of a product, as presented in images.

**Methods:**

The images used the constructs of Simile, Metaphor and Analogy to correspond to congruent, related and incongruent semantic structures, and measured the amplitude of Event-Related Potentials (ERPs) to compare these images with Landscape images. Sixteen participants with design-related educational backgrounds were invited to join in the ERP experiment.

**Results:**

The results found that the image depicting the Metaphor showed a stronger N600 amplitude in the right anterior region of the brain than the Landscape image and the Analogy image induced a stronger N600 effect in the left anterior and right anterior part of the brain than the Landscape image. However, the Simile image did not trigger the N600. The N600 was triggered when the meaning of the Metaphor and Analogy being presented could not be understood. This indicates that a greater processing effort to comprehend them than was required for Simile. Analogy has a wider N600 distribution than Metaphor in the anterior area, suggesting that Analogy would require higher-level thinking processes and more complex semantic processing mechanisms than Metaphor.

**Conclusions:**

The N600 implicated that an assessment method to detect the semantic relationship between appearance and function of a product would assist in determining whether a symbol was suitable to be associated with a product.

## Background

### Figurative design

A product’s design says something about the designer and is therefore a form of communication between the designer and the user; the designer uses symbols that can be understood by potential users to describe the various functions of the product. One of the senses people use to identify and comprehend their external environment is sight but their judgment and their interpretation of what they are seeing will be influenced to a certain extent by their knowledge and experience [1]. The use of Metaphor in design is widely used in product advertising. The abstract symbols designers use to enhance consumers’ perception of the product will become associated with the product and its design. However, it is not certain that every user will interpret the symbols in the same way or as intended. For example, the butterfly chair which designed by Sori Yanagi is such that when seen from the front, the chair looks like a butterfly. However, if the Metaphor of a butterfly is used, the person who wants to sit on the chair may find it difficult to associate the image of a butterfly with that of a chair. This has to do with semantic cognition.

A product can be described in terms of its practical functions, but an additional layer of meaning can be added by the use of Metaphor which may affect the user's perception of the function of the product. Whether people have a background in industrial design or they have no design experience, product recognition is higher than that of design recognition which in turn, is higher than the recognition of correlative meaning [1]. Therefore, if the symbol used to describe the product is not clear or is too abstract, users may well misinterpret the function of the product or its actual shape.

According to Lin and Huang's [1] comprehensive analysis of the semantics used in describing a product, Metaphorical design can be divided into five categories: Metaphor, Simile, allegory, metonymy, and Analogy. This study found that when these are ranked, Simile has the highest recognition rate, followed by Metaphor and Analogy. Metonymy and allegory have the lowest recognition rate. Therefore, this study uses Simile, Metaphor and Analogy as stimuli because they are easily recognized and differences can be noted. Different levels of semantic relevance are offered in images of the product and the functional relevance compared in order to study the extent to which participants associate the semantic structures with the product. Based on Lin andHuang's [1] analysis of the semantics, the meaning of these three categories is explained below.

**Fig. 1:**
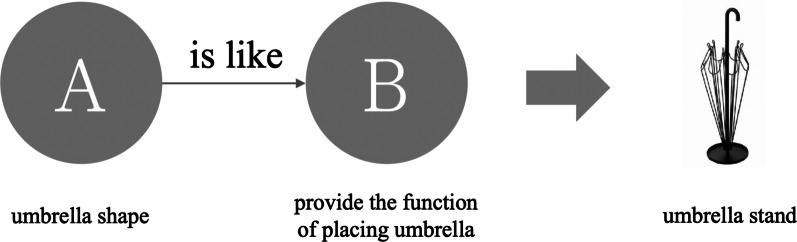
Simile uses "A is B" form and product example (image retrieved from [2])

**Fig. 2:**
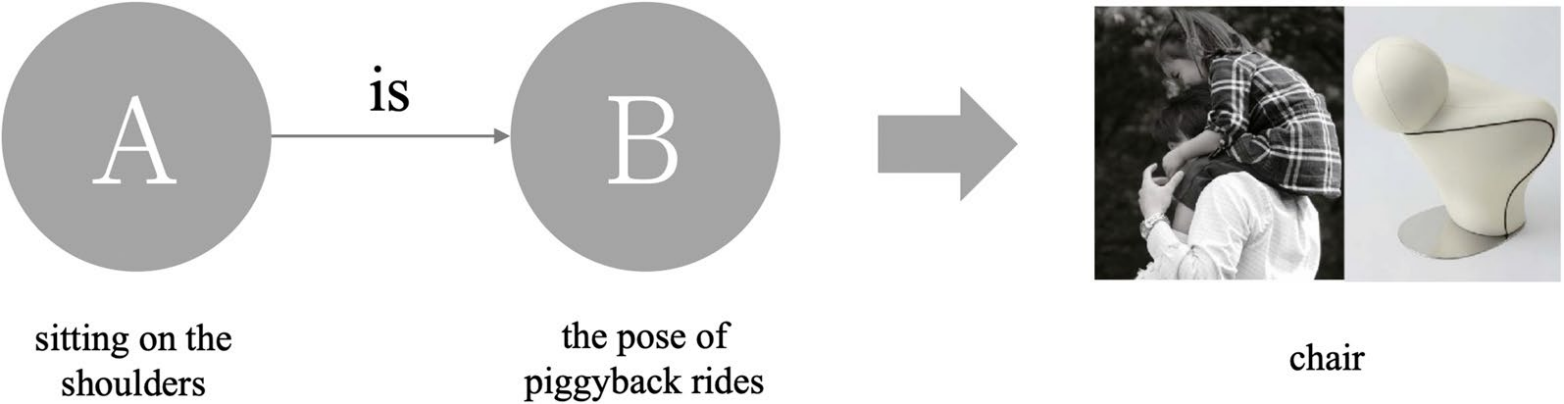
Metaphor uses "A is like B" form and product example (image retrieved from [3])

**Fig. 3:**
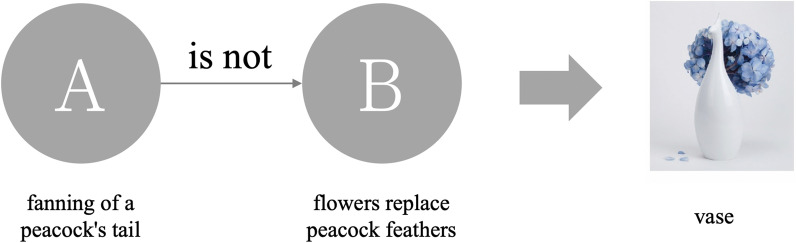
Analogy uses "A is not B" form and product example (image retrieved from [4])

- Simile: Rhetoric is in the form of "A is like B". The product uses the obvious appearance as the symbol of the product. It aims to directly indicate the meaning with no hidden or clear instructions. The design is straightforward and clear as a Simile. Figure 1 is an umbrella stand. The shape uses an umbrella-like outer frame to replace the umbrella function.
- Metaphor: Rhetoric is in the form of "A is B". The product and borrowed symbol have similarities in a certain feature (for example: form, color, and characteristic, etc.). Figure 2 is the Kataguruma chair. The appearance is based on piggyback rides as the symbol of the product. Riding on the shoulders is similar to sitting on a chair. There is a common characteristic of “sitting” between the two. When using the product, it will awaken the memory of riding on the shoulder as a child.
- Analogy: Rhetoric is in the form of "A is not B". It means that there is no direct connection between the product and the borrowed symbol. It associates the meaning of a product with a certain characteristic or situation. Figure 3 shows the Peakco vase, which uses the shape of a peacock to represent the concept of vases and flowers to interpret the functions of the product. However, simply presenting the symbol of a peacock without feathers cannot constitute the association of a vase. Connecting the concept of "flower" with the attribute of "fanning of a peacock's tail" can form a complete association of vases.

Table 1 shows the types of semantic associations between product shape and function in the above three categories. The degree of semantic relevance to these three categories is Simile > Metaphor > Analogy. The degree of semantic comprehension for these three categories is Simile > Metaphor > Analogy. This means that products with higher semantic relevance are easier to understand. Therefore, these three different semantic represent congruent semantic, related semantic, and incongruent semantic, respectively.

**Table 1:** The logic of card sorting

Category	Simile	Metaphor	Analogy
Example			
Appearance	Umbrella stand	Chair	Vase
Borrowed symbols	Umbrella	Piggyback rides	Peacock
Semantic understanding	Easy	Normal	Difficult
Semantic relevance	Congruent	Related	Incongruent

### ERP

People respond emotionally to what they see—this psychological response is closely related to physiological stimuli [5]. Brain waves can be used to measure the psychological reaction to a visual stimulus as different cognitive activities will stimulate different regions in the brain, while the intensity of the potential changes and the distribution will be different. This knowledge can be used to understand and compare the participant’s cognitive processes. Event-Related Potentials (ERPs) (a category of psychophysiology) are electrophysiological signals that can be identified by electroencephalography (EEG): a psychological reaction to a stimulus directly triggers a neural response in the brain. The advantage of an ERP is its high resolution-measurement technology that identifies subtle changes in brain potential over a short period of time. The ability to rapidly record the brain's response to stimuli without the need for external responses means that the cognitive processes of the participants can be studied and inferences made about their responses according to different patterns of neural activity.

#### N400

The N400 (which is a component of ERP) is commonly used to study the principles of language processing. This is a negative waveform that can be measured in the brain after the stimulus has been presented for 300 to 500 ms. It is generally believed that the N400 is related to the extraction of semantic messages in long-term memory and can be used to reflect semantic processing. Some studies have suggested that the comprehension of Metaphor can be measured by the N400 response [6] but there can be difficulties with this in terms of the semantic context of the Metaphor (for example, if the semantic context is weak or absent). In the recording of Metaphors, the N400 manifests different amplitudes according to the degree to which the Metaphor is comprehended and whether it is presented within a recognized semantic context. Coulson and Van Petten [7] found that literal language and Metaphorical language are processed in the same way but that Metaphorical language requires more processing than literal language. Ortiz et al. [8] believe that the use of visual Metaphors can produce the same effect as text, and that Metaphorical images can encourage a stronger N400 response than more familiar images. With familiar images, the N400 response indicated activity in both the left and right hemispheres of the brain, with more in the left side of the brain,when mixed-Metaphor pictures were presented, there was greater activity in the right side of the brain [9–12]. The semantic processing mechanism of familiar images and that of Metaphorical images indicated that mixed Metaphors are different. Because Metaphors use unexpected language, the brain produces more asymmetric activity and stronger current density than when regular language is used.

#### N600

The N600 is a negative wave that is triggered 600–800 ms after the stimulus of inconsistent semantic structures [13–15]. Shibata et al. [16] found that this positive component is similar to the N400 component and can be considered as a second N400 reaction in the later period. Many studies have shown that the N600 is triggered by practical experience and logical thinking or comprehension of the rules as well as inconsistent stimuli such as those provided by humor and entertainment [15, 17]. Therefore, the N600 is often triggered in the detection of humor or jokes, mainly in the observation of N270, N400, N600-800, and even 1500 ms slow waves [14, 17–19].

Samson et al. [20, 21] argue that to comprehend humor, people need to apply the cognitive process of organizational thinking, have insight, and the ability to successfully resolve ambiguous sentences. This is because the understanding of incongruent structures requires more coherent construction and greater psychological manipulation and context reorganization [20] (Chan et al. 2012). Coulson and Kutas [22] indicated that inconsistent stimuli prompted two phases: an early phase where inconsistent stimuli prompted the N400 detection phase, and a later phase where the comprehension of inconsistent stimuli triggered 500–900 ms slow waves. Wang et al. [19], in their study of paintings showing facial humor, found that facial deformation evoked significant differences in the N270, N400, and N600-800 bands, and were indeed related to cognitive dissonance theory.

## Summary

Based on the above ERP literature, N400 component is the response to an inconsistent stimulus; N600 is triggered by the process of a person perceiving an inconsistency and having to re-evaluate the language structure in order to find a solution. This study posits that the semantic processing process of a product is similar to cognitive semantics. Previous studies focused on the relevance of context in the semantics of cognition, such as the semantic similarity of pronunciation (cough–rough), category (cat–dog) and vision (rough–dough). Semantic meaning in both cognition and design explores the "relevance" of semantic meaning, which is based on contextual relevance, and compares the common attributes between them. The difference lies in the input stimulus form. The "meaningful" stimulus may produce ERP effects no matter whether what the non-verbal and verbal forms of text, pictures, sound, and video. Therefore, product pictures as a stimulus should induce ERP effects. The samples of this ERP experiment used actual products to visually explore the correlation between product appearance and category. Based on the relevance of Simile, Metaphor and Analogy in the relationship (see Table 1) between the given semantics and the function of the product the order of the semantic relevance was congruent, related, and incongruent, respectively. According to the degree of semantic association, this study assumes that Analogy might induce the strongest N600 amplitude, followed by the Metaphor. The congruent semantics of Simile should not induce an N600 effect.

## Methods

### Participants

Because the research involves relevant content such as product design, design history, and product knowledge, the participants were required to have a design background. Sixteen participants (ten males, six females; mean age = 23.56 years, Std = 1.32 years) were invited to participate in the experiment. All participants had design-related university degrees, were right-hand dominant and had no presenting adverse conditions such as visual impairment or brain injury. The experimental standards of the study were approved by the China Medical University & Hospital Research Ethics Center of Taiwan (CRREC-109-027). Written informed consent was obtained from all participants prior to participation.

### Materials

#### Simile, Metaphor, and Analogy stimulus

Four experts (male, mean age = 52.75 years, std = 5.9) with over 20 years of teaching and product-design experience were invited to select samples. In the logic of card sorting (see the Table 1), experts judge the semantic comprehension of the product according to the degree of conversion and relevance between the appearance of the product and the borrowed symbol for each category (such as Simile, Metaphor, and Analogy).

**Fig. 4:**
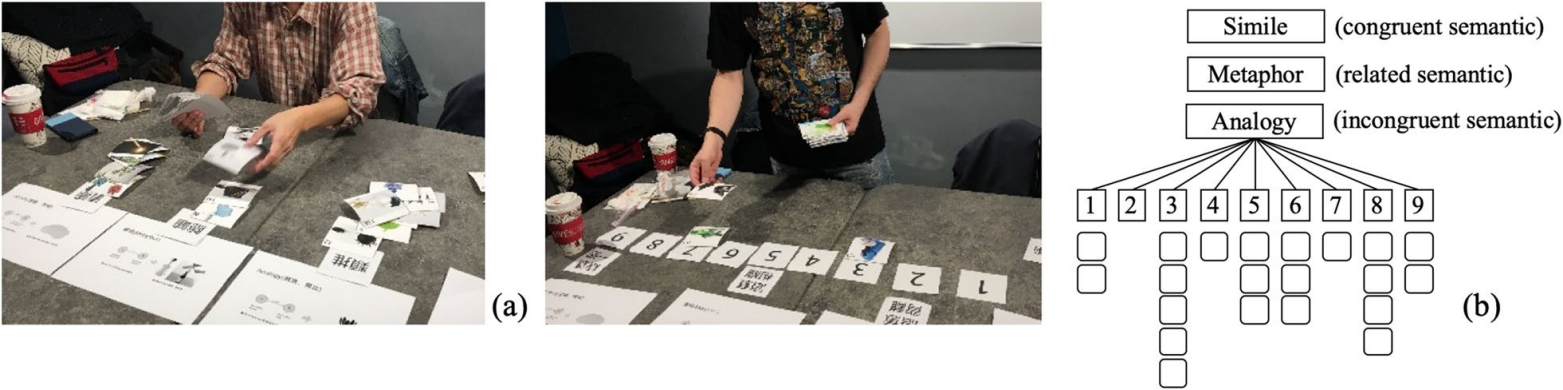
Card sorting: **a** The first step: 160 cards are repeatedly divided into Simile, Metaphor and Analogy categories, and **b** the second step: each category is based on 9 scales of semantic distance (1 = extremely difficult to understand, 9 = extremely easy to understand) for classification. These three categories for semantic understanding represent congruent semantic, related semantic, and incongruent semantic, respectively

The process of card sorting is divided into two stages. In the first stage (Fig. 4a), 160 images were repeatedly divided into 110, 129, and 84 items for the features of the Simile, Metaphor, and Analogy categories. These cards permit more than two categories at this stage. In the second stage (Fig. 4b), each category is classified according to 9 scales of semantic distance (1 = extremely difficult to understand, 9 = extremely easy to understand). The number of pictures in each scale differs or is vacant. If the sample has more than two categories, the experts will discuss and decide which category it belongs to. A total of 120 images were screened (see the “Appendix 1”). The averages of the three categories (see the Table 2) were 4.63, 5.19, and 3.71, respectively. If the expert is not clear about the content of the picture during the classification process, the researcher will explain it to the expert but will not guide him to make a choice.

**Table 2:** Classification results of simile, metaphor and analogy

	Simile	Metaphor	Analogy
The original number	160	160	160
The number of first grouping	110	129	84
The number of second grouping	40	40	40
Mean	4.63	5.19	3.71
SD	3.60	3.64	3.81

#### Neutral stimulus

**Fig. 5:**
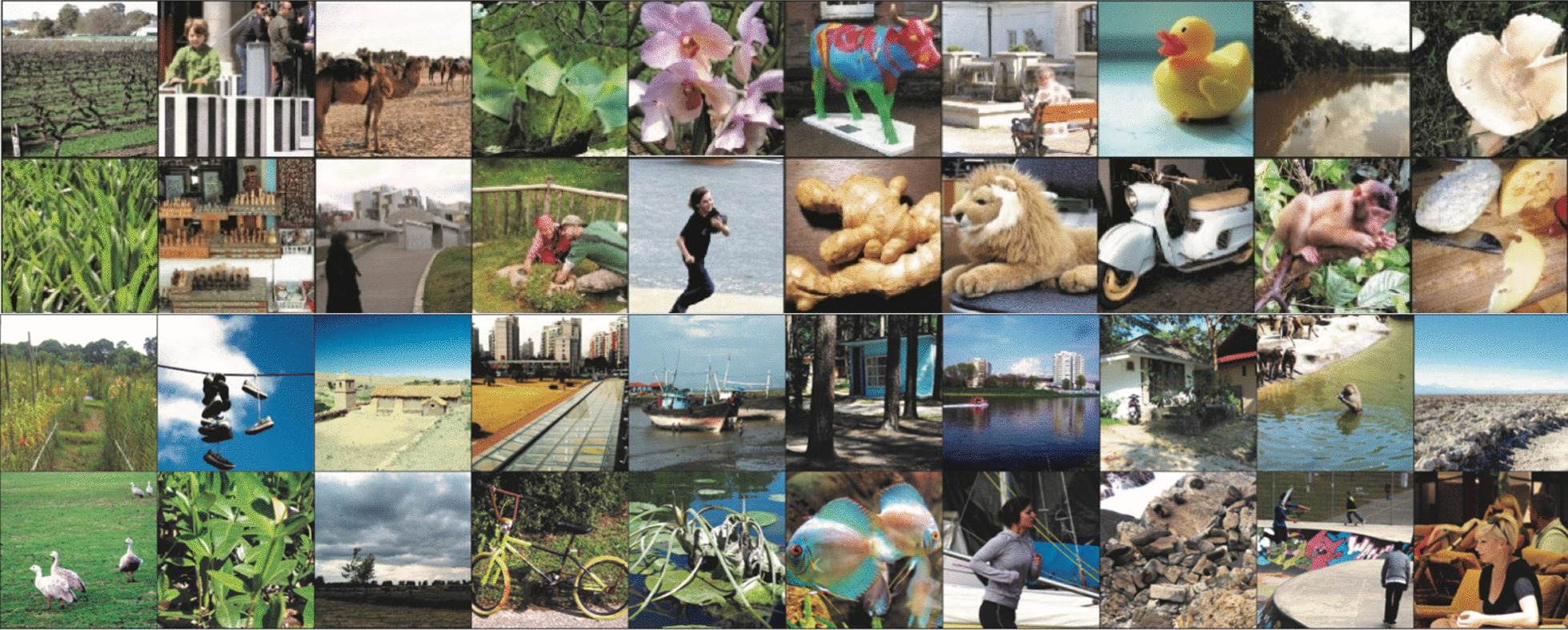
Neutral stimulus

In addition, 40 colorful neutral images (Fig. 5) were selected from the Nencki Affective Picture System (NAPS), including: Landscapes, animals, plants, artifactual objects, buildings, roads, and humans [23–26]. These stimuli provided a baseline for comparisons among Simile, Metaphor, and Analogy stimulus.

### ERP recording

The equipment used in this study was a 32-channel Bluetooth wireless physiological feedback device: NeXus-32 (Mind Media, The Netherlands). All channels were referenced to an electrode located between Fz and FCz, and were re-referenced off-line to the average of the two mastoid electrodes [27–30]. The electrode impedance was 5 kΩ, the brain wave recording frequency was 250 Hz, and the data was filtered at 0.5–60 Hz [31]. Noise was filtered out by means of BioTrace + software, with the maximum amplitude (over 80 μV) eye movement signal per signal [32, 33], and frontal muscle signal with frequency greater than 20 Hz [34] was used as the standard. A baseline correction (− 100 to 0 ms) was applied. The brain wave data elicited in the experiment was analyzed and processed using WINEEG software. The continuously recorded brain waves were segmented for a total period of 0–1000 ms and the EEG data was converted to ERP format. The time windows for the N400 and N600 were 300–500 ms and 600–748 ms, respectively. Then, the brainwave segments of each participant were overlaid and averaged. Finally, the brainwave amplitude of all the participants was averaged for statistical analysis.

### Procedure

Participants were fitted with an elastic electrode cap and then seated in an electrically isolated chamber. The participants were asked to sit approximately 70 cm from the screen. After confirming that the participants clearly understood the process, the experiment began with a total of 160 picture-word combinations, including figurative products (Simile, Metaphor, and Analogy) (see the “Appendix 2”) and neutral images. Each word in Chinese was paired with a picture. In order for the meaning of the presented words to be easily understood by the participants and for them to quickly enter the topic, the naming method of each word must be simple and 2–4 Chinese words. Among the words, the 120 trials of prime words for the Simile, Metaphor, and Analogy images appeared as the "product’s category" (i.e., "椅子(chair)", "燈(lamp)", "廚具(kitchenware)", "文具(stationery) ", etc.). There were 62 product categories in total. In addition, the 40 trials of prime words for the neutral images showed "風景(Landscape)".

**Fig. 6:**
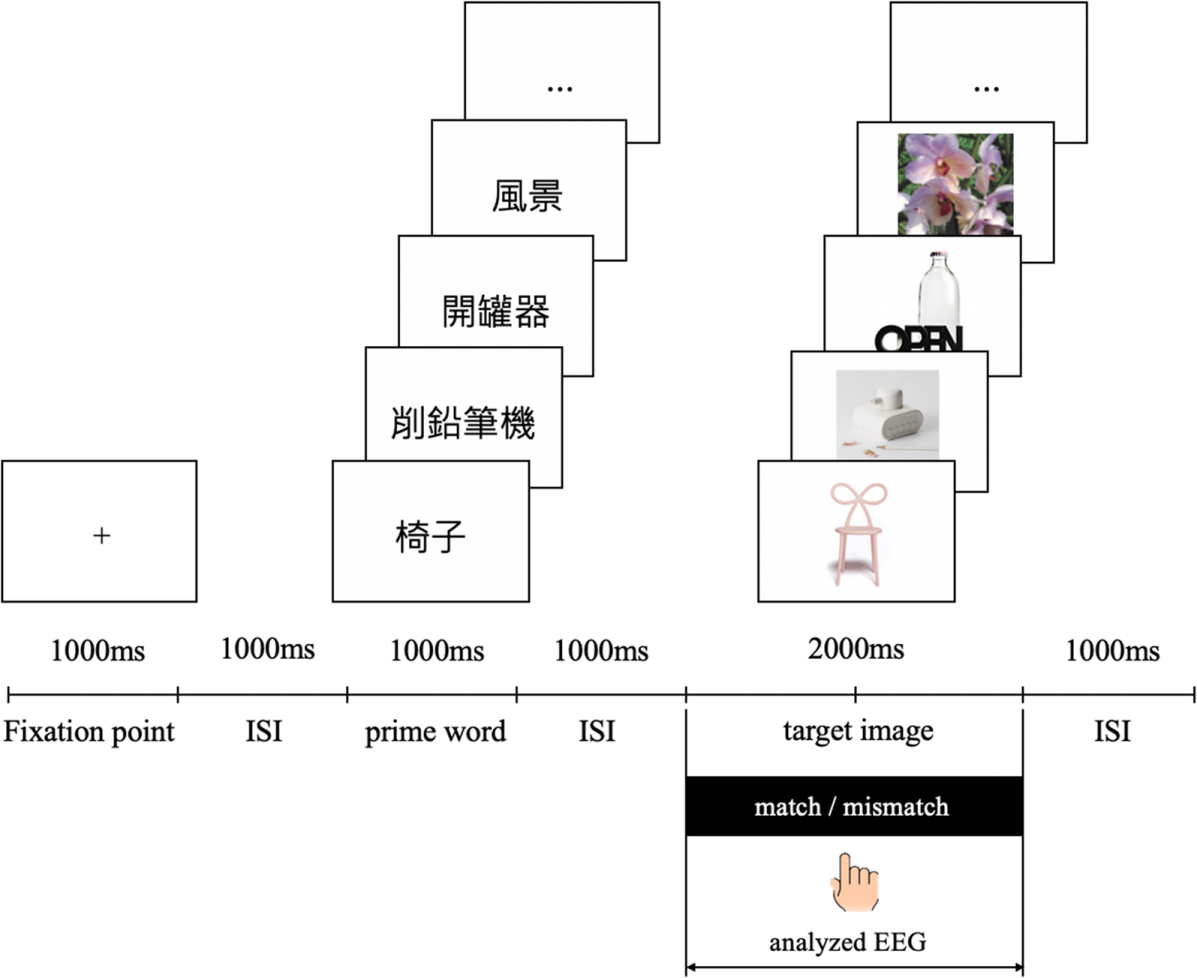
Procedure

Figure 6 shows the ERP Procedure. Each trial began by showing a fixation point at the center of the screen for 1000 ms. After the fixation point had disappeared, the prime word in Chinese (i.e., "椅子(chair)") was presented for 1000 ms, followed by a blank screen for 1000 ms. The target then appeared and remained on the screen for 2000 ms. During this period, the participants were required to judge whether the symbol of the shape (appearance) of the target matched the product category expressed in the preceding word. The target is a chair with a butterfly-shaped back, and its appearance differs from that of ordinary chairs. The participants compared typical chair types they imagined (i.e., such as four legs and a platform for sitting) to compare with the various products they saw in this experiment. Participants were told to press a button with the index finger of one hand to register a response match, and to register non-matches with the index finger of the opposite hand. To avoid their dominant hand influencing the match/mismatch response, the assignment of the fingers to the response categories was counterbalanced across participants: half of the participants used their right index finger to input "match", and the other participants used it to input "mismatch". The screen then went blank for 1000 ms, and the next trial started. There was a ten-minute break after 160 trials. Each word and image were presented once in random order. The EEG signal was recorded throughout the experiment. Among the results, the analyzed EEG was extracted from the interval of target appeared and remained on the screen for 2000 ms.

### ERP data analysis

**Fig. 7:**
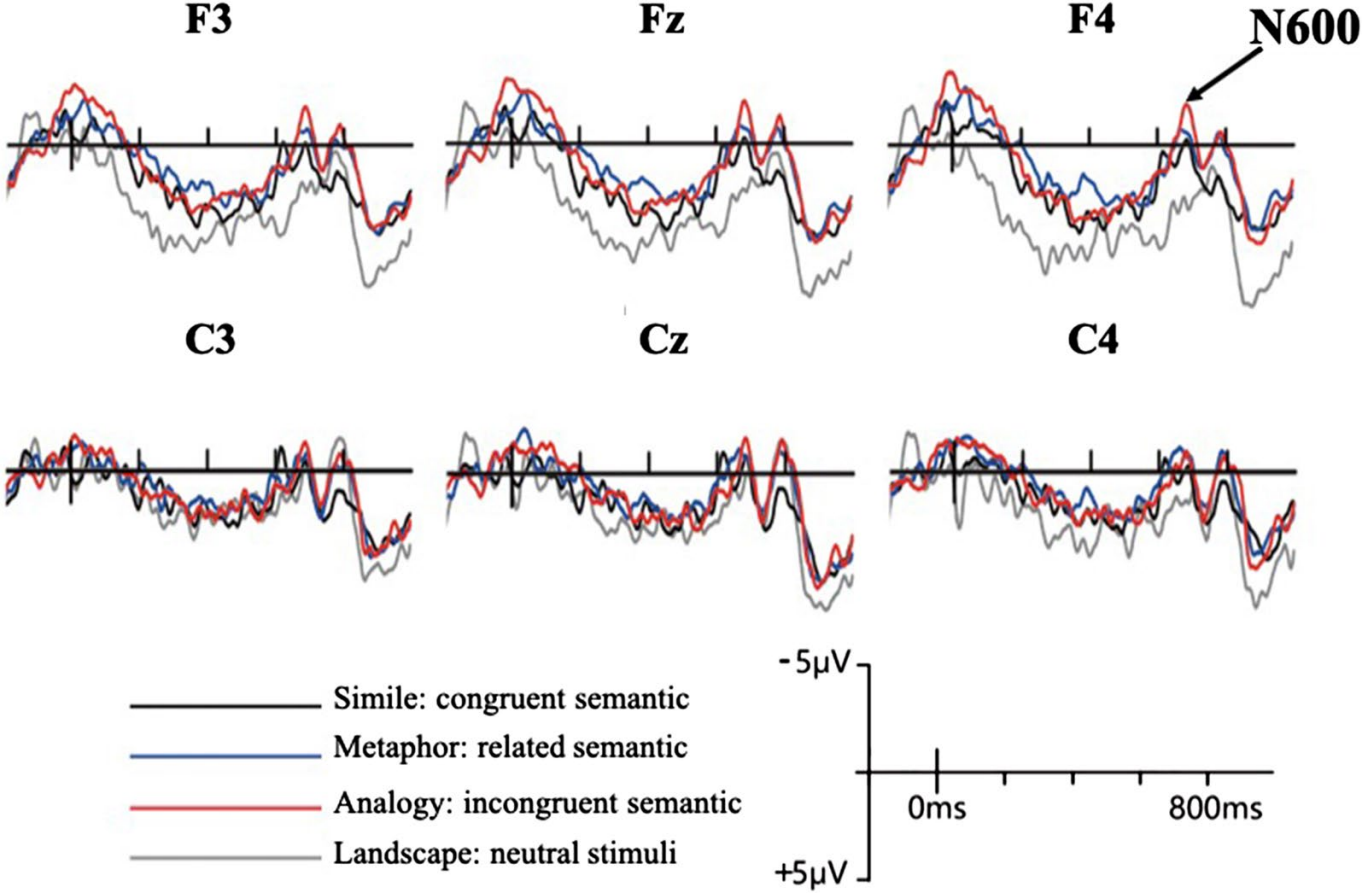
ERP waveforms

**Fig. 8:**
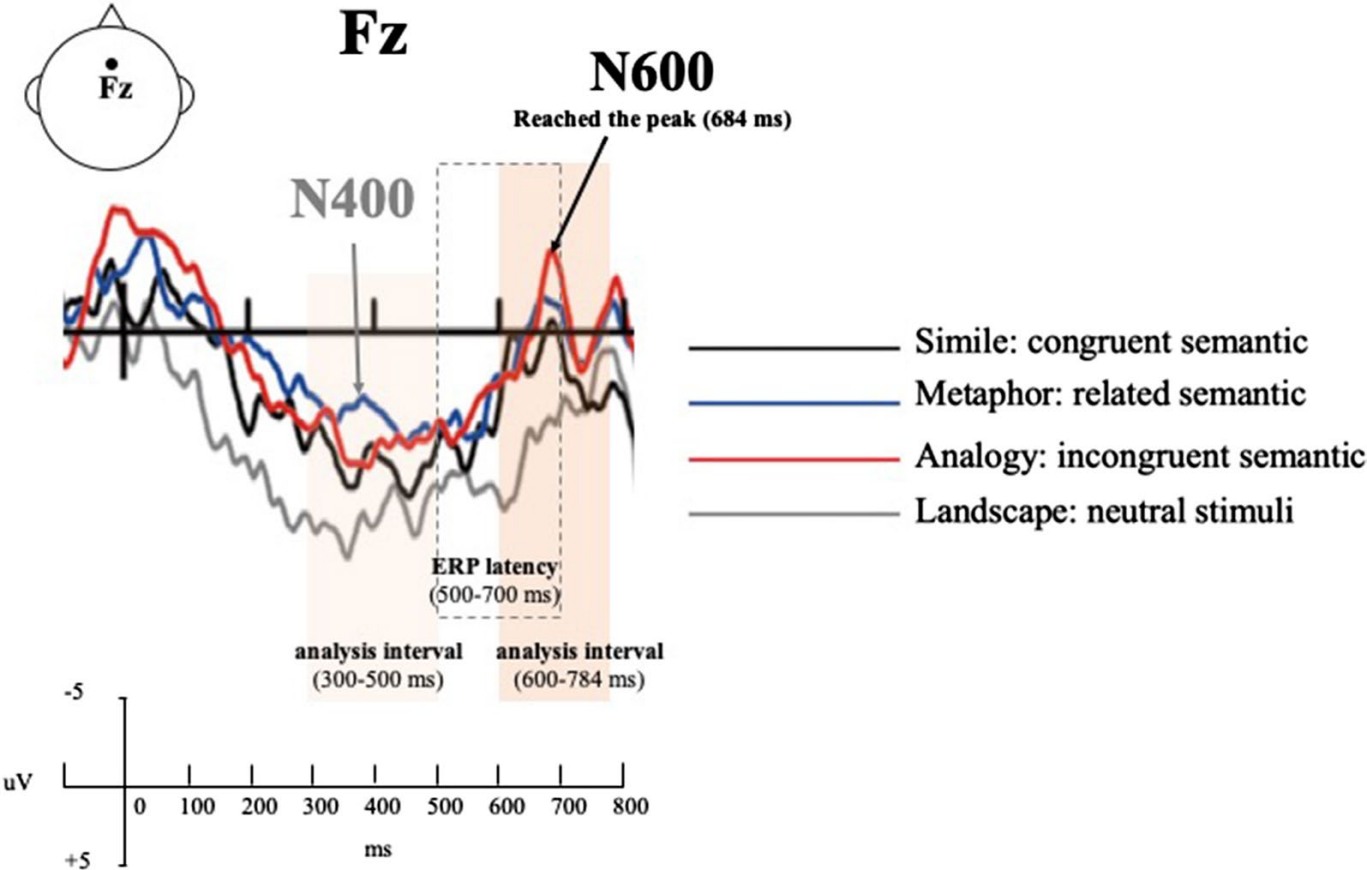
Ground average ERP: F3, Fz, F4, C3, Cz, and C4 electrode sites were selected and analyze N400 (300–500 ms) and N600 (600–748 ms) effects

The selection of the electrode sites (F3, Fz, F4, C3, Cz, and C4) (Fig. 7) was based on previous studies [35–38]. Krawczyk [39] found that N600 mainly appears in the prefrontal cortex. Figure 8 shows the ERP waveforms of F3, Fz, F4, C3, Cz and C4. The waveform direction of all stimuli developed at approximately 80 ms and became negative at about 600 ms. The N600 appeared between 600 and 748 ms, peaking at 684 ms (Fig. 8). Therefore, the N600 component was selected and the time window was averaged at this interval (yellow area) after target onset. This section was based on recommendations made in previous N600 research [17, 19, 22]. However, there is no obvious 400 waveform over the 300–500 ms time period after stimulus onset. Several previous N400 studies [40–42] also informed the selection of the N400 measurement interval.

Repeated measures ANOVA using SPSS statistics 24 software was used to understand whether there was a difference in ERP based on three different semantic categories between product appearance and borrowed symbols, including congruent semantic, related semantic, and incongruent semantic. This experiment was the within-subjects study design, i.e., the same person tests all the conditions.

**Fig. 9:**
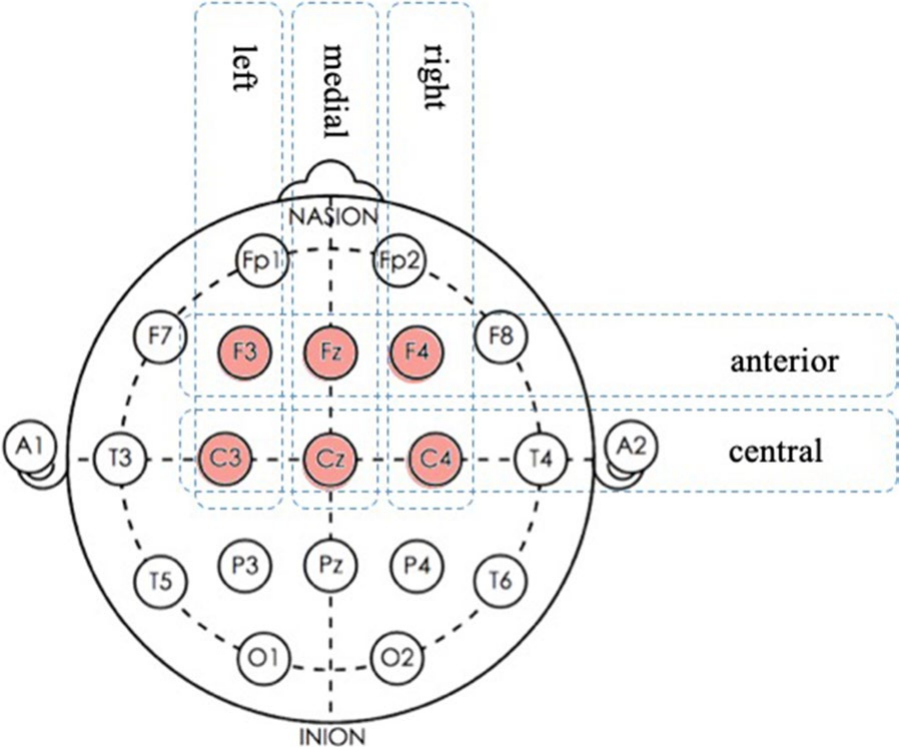
The six electrode sites were divided into "anterior-central" and "left-medial-right" positions

MANOVA analysis were divided into two steps. The first three-way MANOVA analysis factor included the “category” (Simile, Metaphor, Analogy, and Landscape), and the electrode sites (Fig. 9) of the “anterior-central” and “left-medial-right” positions (left anterior: F3; medial anterior; Fz, right anterior: F4; left central: C3; medial central: Cz; right central: C4). The Greenhouse–Geisser correction for non-sphericity was applied where appropriate. Further analysis was used the paired sample *t*-test to ensure any potentially important factors when the interaction was significant.

## Results

### Behavioral results

Table 3 displays the percentages of match responses for Simile, Metaphor, Analogy, and Landscape: 82.19%, 73.28%, 55.63%, and 99.22%, respectively. The levels of difficulty of the semantic matching task were Landscape, Simile, Metaphor, and Analogy in order from the easiest to the most difficult. The behavioral results of the four categories verified the semantic associations between product shape and function in the forenamed Table 1.

**Table 3:** Behavioral results for four categories in the semantic judgment tasks (standard deviation of mean in parentheses)

Prime word-target image	Match response	
Simile	82.19%	(5.15%)
Metaphor	73.28%	(6.10%)
Analogy	55.63%	(8.29%)
Landscape	99.22%	(1.51%)

### N400 (300–500 ms) effect

Table 4 shows the average amplitudes of N400 for Simile, Metaphor, Analogy, and Landscape: 1.70, 1.08, 1.88, and 1.40 μV, respectively. In terms of numerical values, the amplitude of N400 on the four categories from largest to smallest were Analogy, Simile, Landscape, and Metaphor.

**Table 4:** The average amplitudes of N400 for simile, metaphor, analogy, and landscape (standard deviation of the mean in parentheses) (unit: μV)

Category	Left anterior	Medial anterior	Right anterior	Left central	Medial central	Right central	All
Simile	2.19	2.20	2.44	1.10	0.98	1.31	1.70
	(3.87)	(3.98)	(3.91)	(1.95)	(2.26)	(1.99)	(2.99)
Metaphor	1.37	1.40	1.28	0.93	0.80	0.69	1.08
	(2.71)	(2.80)	(2.52)	(1.74)	(1.86)	(1.68)	(2.22)
Analogy	2.29	2.36	2.62	1.33	1.42	1.26	1.88
	(3.18)	(3.16)	(3.28)	(1.99)	(2.22)	(2.06)	(2.65)
Landscape	1.57	1.79	2.39	0.54	0.69	1.40	1.40
	(2.73)	(2.77)	(2.97)	(1.80)	(1.95)	(1.72)	(2.32)

Table 5 presents the overall result of MANOVA analysis of N400 for the category, anterior-central, and left-medial-right factors. Only the "anterior-central" position factor has the main effect (F[1,30] = 5.93, *p* = 0.03, ε = 1). However, there were no interactions between the "category" x "anterior-central" position factors (F[3,45] = 1.03, *p* = 0.39) and "category" x "left-medial-right" position factors (F[6,90] = 2.29, *p* = 0.07).

**Table 5:** The overall result of MANOVA analysis of N400 for the category, anterior-central, and left-medial-right factors

Item	SS	*df*	MS	F	Sig
Category	35.74	3	18.91	0.84	0.44
Anterior-central	87.44	1	87.44	5.93	0.03*
Left-medial-right	5.06	2	3.65	2.84	0.16
Category × anterior-central	5.54	3	1.85	1.03	0.39
Category × left-medial-right	9.85	6	2.58	2.29	0.07
Anterior-central × left-medial-right	0.30	2	0.23	0.34	0.62
Category × anterior-central × left-medial-right	0.84	6	0.24	0.46	0.75

*p < .05

### N600 (600–748 ms) effect

Table 6 shows the average amplitudes of N600 for Simile, Metaphor, Analogy, and Landscape: 0.63, 0.10, 0.03, and 1.17 μV, respectively. In terms of numerical values, the amplitude of N600 on the four categories from largest to smallest were Analogy, Metaphor, Simile, and Landscape.

**Table 6:** The average amplitudes of N600 for simile, metaphor, analogy, and landscape (standard deviation of the mean in parentheses) (unit: μV)

Category	Left anterior	Medial anterior	Right anterior	Left central	Medial central	Right central	All
Simile	0.81	0.78	0.85	0.47	0.45	0.44	0.63
	(1.95)	(2.11)	(2.14)	(1.27)	(1.31)	(1.11)	(1.65)
Metaphor	0.24	0.00	0.19	0.20	− 0.04	0.03	0.10
	(2.18)	(2.33)	(2.46)	(1.09)	(1.24)	(1.21)	(1.75)
Analogy	− 0.05	− 0.11	− 0.05	0.13	0.07	0.20	0.03
	(2.10)	(2.23)	(2.22)	(1.21)	(1.31)	(1.24)	(1.72)
Landscape	1.65	1.71	2.09	0.31	0.41	0.86	1.17
	(3.62)	(3.98)	(4.11)	(2.10)	(2.36)	(2.32)	(3.08)

Table 7 presents the overall result of MANOVA analysis of N600 for the category, anterior-central, and left-medial-right factors. The "left-medial-right" position factor has the main effect (F[2,30] = 3.29, *p* = 0.05, ε = 0.77). Moreover, there was an interactions between the "category" x "anterior-central" position factors (F[3,45] = 5.31, *p* = 0.003, ε = 0.54). Moreover, the "category" x "left-medial-right" position factors exhibit an interaction (F[6,90] = 3.62, *p* = 0.003, ε = 0.52).

**Table 7:** The overall result of MANOVA analysis of N600 for the category, anterior-central, and left-medial-right factors

Item	SS	*df*	MS	F	Sig
Category	76.97	3	47.07	2.20	0.14
Anterior-central	12.64	1	12.64	2.11	0.17
Left-medial-right	1.89	2	0.95	3.29	0.05*
Category × anterior-central	30.02	3	18.50	5.31	0.02*
Category × left-medial-right	3.30	6	1.06	3.62	0.02*
Anterior-central × left-medial-right	0.04	2	0.02	0.10	0.90
Category × anterior-central × left-medial-right	0.27	6	0.12	0.47	0.65

*p < .05

Further analysis on *t*-test for Landscape versus Metaphor discovered that only the N600 amplitude of the "right anterior" position was the highest (t[15] = 2.08, *p* = 0.053). Moreover, Landscape versus Analogy for *t*-test results found that the N600 had the highest effect in the "left anterior" and "right anterior" positions (t[15] = 2.14, *p* = 0.05 and t[15] = 2.27, *p* = 0.04, respectively). Simile versus Metaphor for *t*-test results showed that the N600 had a significant difference in the "right central" position (t[15] = 2.09, *p* = 0.05) (Table 8). Also, Simile versus Analogy for *t*-test results displayed the highest N600 effect in the "right anterior" position (t[15] = 2.08, *p* = 0.05). However, there was no significant difference in the N600 amplitudes for Landscape versus Simile and Metaphor versus Analogy. The results showed that the metaphor and analogy did have larger N600 amplitudes than Simile.

**Table 8:** The overall result of paired sample *t*-test of N600 for simile, metaphor, analogy, and landscape

Paired comparison	T	*df*	Sig
Landscape versus simile			
Left anterior	1.04	15	0.32
Medial anterior	1.10	15	0.29
Right anterior	1.44	15	0.17
Left central	− 0.26	15	0.80
Medial central	− 0.06	15	0.96
Right central	0.81	15	0.43
Landscape versus metaphor			
Left anterior	1.65	15	0.12
Medial anterior	1.92	15	0.07
Right anterior	2.08	15	0.05*
Left central	0.21	15	0.84
Medial central	1.00	15	0.33
Right central	2.01	15	0.06
Landscape versus analogy			
Left anterior	2.14	15	0.05
Medial anterior	2.07	15	0.06
Right anterior	2.27	15	0.04
Left central	0.45	15	0.66
Medial central	0.62	15	0.55
Right central	1.25	15	0.23
Simile versus metaphor			
Left anterior	1.52	15	0.15
Medial anterior	1.74	15	0.10
Right anterior	1.62	15	0.13
Left central	1.14	15	0.27
Medial central	1.82	15	0.09
Right central	2.09	15	0.05
Simile versus analogy			
Left anterior	1.86	15	0.08
Medial anterior	1.86	15	0.08
Right anterior	2.08	15	0.05
Left central	1.45	15	0.17
Medial central	1.31	15	0.21
Right central	0.99	15	0.34
Metaphor versus analogy			
Left anterior	0.76	15	0.46
Medial anterior	0.42	15	0.68
Right anterior	0.66	15	0.52
Left central	0.53	15	0.61
Medial central	− 0.42	15	0.68
Right central	− 0.93	15	0.37

*p < .05

### Topography

**Fig. 10:**
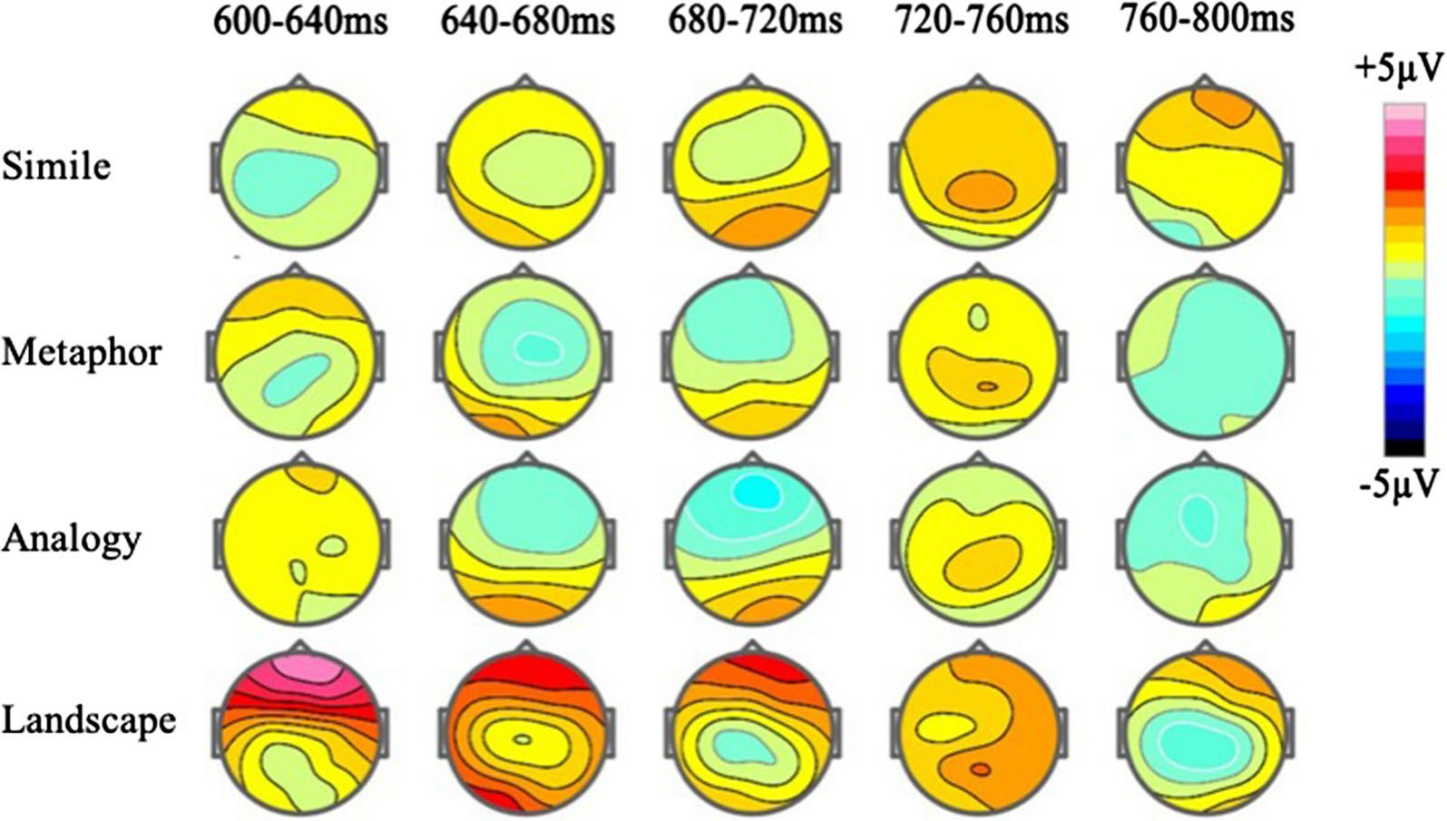
Topography: The distribution of brains in the N600 effect for the Simile, Metaphor, Analogy, and Landscape images over the 600–800 ms time window

The Metaphor and Analogy respectively compare the stimuli of the Landscape, and both have significant N600 effects in the anterior area. Therefore, it can be observed from topography. Figure 10 shows the overall topography of the brain over the 600–800 ms time window. From the 640–720 ms time window, Metaphor and Analogy have a distinct N600 amplitude distribution (blue block) in the frontal region of the brain. Of these, the N600 effect from Metaphors in the 640–680 ms time window tends to manifest in the anterior region of the right side of the brain. Analogy has a more widely distributed N600 effect in the frontal lobe of both the left and right sides of the brain. However, Simile triggers no significant N600 effect. In addition, the amplitude of the images of the Landscape was positively reflected in the anterior region (red block), as was expected.

## Discussion

This study investigated the impact of semantic relevance on the ability to comprehend images of the appearance and function of a product. Pictures showing Simile, Metaphor, and Analogy that corresponded to congruent, related, and incongruent semantic structures were presented and the amplitude of ERP was measured to compare the differences of these with an neutral image. It was found that the Metaphor image elicited a larger N600 amplitude in the right anterior region of the brain than the Landscape image and that the Analogy image induced a stronger N600 effect in the left anterior and right anterior regions than the Landscape image. However, the Simile image did not give a different N600 when compared to the neutral picture.

### Category consistency did not trigger the N400 response

The Simile, Metaphor, and Analogy images did not trigger an N400 response, indicating that participants could identify these categories. In general, if pictures of different categories (such as books and hammers) were combined with pictures of the same category (such as zebras and white horses), a higher N400 was triggered [42–45]. Because the images in this study all belonged to the same category and there was thus no semantic conflict, it was expected that the N400 effect would not be generated. The participants in this study could detect the semantic relationship between the appearance of the product and its function, which made it easy for them to identify the image. However, once the participant was unable to understand the correlation between the stimulus, the late N600 slow wave was triggered.

### Incomprehensible images triggered an N600 effect

The N600 is a waveform that is generated when semantic inconsistency is detected and the person makes an effort to deal with the inconsistency [15, 17]. In this study, the ranking of the N600 amplitude from highest to lowest showed Analogy > Metaphor > Simile. This result is consistent with Lin and Huang's [1] study, indicating that the order of Metaphorical design interpretation was Simile > Metaphor > Analogy. This suggests that semantic structures using Metaphor and Analogy are not as easily understood as Simile. Simile is a direct design method that has no hidden meaning and gives clear indications of meaning [1]. Therefore, Simile is intuitive and easy to comprehend, and there is little semantic conflict. As expected, Simile triggered no N600 in this study. Metaphor and Analogy present ambiguous semantic structures, even if there is a slight semantic association between the product’s appearance and function. Regardless of their semantics, which was related to introspection, those who did not have any background knowledge or understood the meaning of the images, needed to spend time trying to make sense of what they had seen after the viewing.

The results obtained in this study indicate that the images involving Metaphor and Analogy triggered the N600 response; thus, the participants were engaging in a process of comprehending the unfamiliar semantics. This contradicts previous research which has suggested that the use of Metaphor should trigger an N400 response [6, 7]. Other studies have confirmed that an N600 response could follow the second N400 response in the later period [16], indicating that N600 was a slow wave, and the mechanism of processing various semantic structures should have some commonality.

Some characteristics of Metaphor are similar to borrowed symbols (e.g., morphology, color, nature, etc.) [1]. The borrowed symbols could have been hidden in the expression of the appearance and therefore not easily noticeable. However, there is no direct correlation between Analogy and borrowed symbols [1] and any connection needs to be made by discerning common attributes and integrating these by means of past experience [46–48]. It could be seen that the level of semantic relevance and associations made in Analogy was more complex than that of discerning the meaning of Metaphor. Therefore, the N600 amplitude triggered by the image depicting Analogy was stronger than that for the image showing Metaphor.

### Differences in activity between related and incongruent semantic structures in various regions of the brain

The N600 response to Analogy showed a wider distribution than it did to Metaphor in both the left- and right anterior regions. The response to Metaphor was mainly concentrated in the right anterior region. Research has shown that the N600 waves are most active in the frontal cortex [35–38]. This was confirmed by the results of this study which found that the N600 effect was induced in the anterior part of the brain. Because the images showing Metaphor offered unexpected concepts for ordinary items, interpreting these requires greater semantic processing and therefore, more intense activity and density in the brain [8]. The effect of images showing Metaphor was N600 distribution in the right anterior region of the brain while images depicting Analogy caused N600 distribution in the anterior area. This suggests that images depicting Analogy should require higher-level thinking and more complex semantic processing mechanisms than images depicting Metaphor [49, 50].

## Conclusion

This study used images showing Simile, Metaphor, and Analogy to detect semantic associations between the appearance and function of a product and to measure ERP. ERP has the advantages of high-resolution measurement technology, which enables us to explore the cognitive process of participants in depth, and to compensate for the authenticity of the questions and responses of the questionnaires from an objective perspective. Simile, Metaphor, and Analogy were distinguished by the card-sorting method and classification according to semantic relevance: congruent, related and incongruent semantic structures. The results indicated by the N600 could prove that the relationship between pictures depicting Metaphor and Analogy and borrowed symbols was indirect or even not related, and both required semantic reassessment to be understood. However, the association between the pictures showing Simile and symbols was straightforward and did not require detailed interpretation.

The results detected significant ERP effects. Although the application of ERP has not been used much as a form of measurement in the design field, further use may be a way to help design research and expand new methods. The results of this study promoted the application of ERP methods to design research and helped physiology researchers understand more about the function of N600.

As was proposed, the main aims for the use of ERPs for this study were: (1) to improve the typicality of neutral stimuli. The content of the study using Landscape images as the neutral stimuli contained elements of views, people and animals, but the content of these pictures was complex. This may have overstimulated certain regions of the brain and triggered other emotions, resulting in higher ERP amplitudes. It is recommended that in the future, a gray image be used as the neutral stimulus, to reduce additional irritation. (2) It is also recommended that the variability of the stimuli in the N600 be considered. The same category of stimulus should be selected (such as chairs or lamps), and very different pictures in terms of appearance and structure be selected. This would make it easier to compare the differences in semantic relevance. (3) Participants with high sensitivity and understanding of the product should be selected. Although this study selected participants who had a background in design, there were cognitive differences in product design. It is recommended that that participants complete a fitness test (including thinking and sensitivity surveys) before the experiment and that suitable participants are selected. This would reduce cognitive differences.

## Data Availability

The datasets generated and/or analysed during the current study are available in the [Google Docs] repository, [https://reurl.cc/7rzeo9].

## Appendix 2: Picture-word matching combination for the simile, metaphor, and analogy images

See Tables 9, 10, and 11.

**Table 9:** Picture-word matching combination of simile images

Prime word (Chinese/English)		No.	Prime word (Chinese/English)		No.
開瓶器	Bottle opener	20	調味罐	Spice jar	105
門鈴	Doorbell	149	隔熱墊	Heat proof mat	61
鬧鐘	Alarm clock	152	時鐘	Clock	99
廚具	Kitchenware	46	衣架	Hanger	87
垃圾箱	Trash can	75	咖啡機	Coffee maker	150
燈具	Lamp	4	打火機	Lighter	151
靜止的	Stationery	160	澆水器	Watering can	11
揚聲器	Speaker	147	訂書機	Stapler	58
隨身碟	Flash drive	148	餐巾架	Napkin holder	91
喇叭	Loudspeaker	25	沙發	Sofa	118
時鐘	Clock	84	削鉛筆機	Pencil sharpener	69
水塞	Water plug	104	燈具	Lamp	102
餐具	Tableware	77	手工具	Handle tool	74
廚具	Kitchenware	126	削鉛筆機	Pencil sharpener	113
雨傘架	Umbrella stand	37	隔熱墊	Heat proof mat	63
椅子	Chair	79	肥皂盒	Soap dish	83
膠帶	Tape	128	筆筒	Pen holder	72
手機座	Phone holder	89	磁鐵	Magnet	82
喇叭	Loudspeaker	154	花瓶	Vase	115
燈具	Lamp	144	醬料碟	Sauce dish	129

**Table 10:** Picture-word matching combination of metaphor images

Prime word (Chinese/English)		No.	Prime word (Chinese/English)		No.
紙捲	Paper roll	124	便條	Note	42
門擋	Doorstop	24	鑰匙扣	Key holder	18
鑰匙扣	Key holder	19	廚具	Kitchenware	30
訂書機	Stapler	22	夾子	Clip	80
書擋	Bookend	35	書籤	Bookmarks	142
文具	Stationery	23	打火機	Lighter	92
門鈴	Doorbell	66	廚具	Kitchenware	36
杯墊	Coaster	60	澆水器	Watering can	90
削鉛筆機	Pencil sharpener	28	梳子	Comb	155
椅子	Chair	2	儲物盒	Storage box	62
書擋	Bookend	49	紙捲	Paper roll	108
燈具	Lamps	12	蛋架	Egg holder	109
水塞	Water plug	97	椅子	Chair	3
夾子	Clip	40	存錢筒	Piggy bank	43
桌子	Table	107	削鉛筆機	Pencil sharpener	78
筆記	Note	110	訂書機	Stapler	153
盆栽	Pot culture	121	筆筒	Pen holder	86
調味罐	Spice jar	100	沙發	Sofa	114
醬料碟	Sauce dish	94	廚具	Kitchenware	55
鑰匙扣	Key holder	106	手工具	Handle tool	131

**Table 11:** Picture-word matching combination of analogy images

Prime word (Chinese/English)		No.	Prime word (Chinese/English)		No.
削鉛筆機	Pencil sharpener	137	澆水器	Watering can	159
牙籤罐	Toothpick pot	7	文具	Stationery	34
燈具	Lamp	95	水塞	Water plug	52
門擋	Doorstop	9	椅子	Chair	134
掛鈎	Hook	13	調味罐	Spice jar	26
隔熱墊	Heat proof mat	53	湯勺	Ladle	56
花瓶	Vase	135	訂書機	Stapler	70
檯燈	Lamps	31	文具	Stationery	81
收線器	Reel	71	燈具	Lamps	145
靜止的	Stationery	120	凳子	Stool	146
茶壺	Teapot	59	凳子	Stool	127
固定器	Holder	29	榨汁機	Juicer	67
凳子	Stool	136	花瓶	Vase	103
屏風	Screen	88	夾子	Clip	85
花瓶	Vase	96	筷架	Chopstick holder	156
盆栽	Pot culture	119	靜止的	Stationery	51
花瓶	Vase	45	掛鈎	Hook	41
掛鈎	Hook	130	拖鞋	Flip flop	111
掛鈎	Hook	132	膠帶分配器	Tape dispenser	47
餐具	Tableware	116	燈具	Lamp	133
